# Non-Native Conformational Isomers of the Catalytic Domain of PCSK9 Induce an Immune Response, Reduce Lipids and Increase LDL Receptor Levels

**DOI:** 10.3390/ijms19020640

**Published:** 2018-02-24

**Authors:** Chuantao Jiang, Hersharan Nischal, Hua Sun, Li Li, Ying Cao, Peng Wei, Jui-Yoa Chang, Ba-Bie Teng

**Affiliations:** 1 Research Center for Human Genetics at Brown Foundation Institute of Molecular Medicine, The University of Texas Health Science Center at Houston, 1825 Pressler St., Houston, TX 77030, USA; chuantao41@yahoo.com (C.J.); nischalsharan04@gmail.com (H.N.); hua.sun@uth.tmc.edu (H.S.); rowenjychang@gmail.com (J.-Y.C.); 2 Research Center for Precision Biomedicine at Brown Foundation Institute of Molecular Medicine, The University of Texas Health Science Center at Houston, 1825 Pressler St., Houston, TX 77030, USA; Li.li@uth.tmc.edu; 3 Department of Biostatistics, The University of Texas MD Anderson Cancer Center, 1400 Pressler St., Houston, TX 77030, USA; yingcao6@gmail.com (Y.C.); pwei2@mdanderson.org (P.W.); 4 Consortium on Aging, The University of Texas Health Science Center at Houston, Houston, TX 77030, USA; 5 The University of Texas MD Anderson Cancer Center UTHealth Graduate School of Biomedical Sciences, Houston, TX 77225, USA

**Keywords:** PCSK9, scrambled disulfide bonds, cholesterol, triglyceride, LDL receptor

## Abstract

PCSK9 (Proprotein convertase subtilisin/kexin type 9) increases plasma cholesterol levels by promoting LDL receptor degradation. Current antibody inhibitors block the interaction between PCSK9 and LDL receptors, significantly decrease plasma cholesterol levels, and provide beneficial clinical outcomes. To reduce the action of PCSK9 in plasma, a novel strategy that will produce a panel of non-native, conformationally-altered isomers of PCSK9 (X-PCSK9) to develop active immunotherapy targeting of native PCSK9 and inhibiting/blocking the interaction of PCSK9 with LDL receptor, thus decreasing plasma cholesterol levels is proposed. The authors used the scrambled disulfide bond technique to generate conformationally-altered isomers of the catalytic domain of mouse PCSK9. The focus was on the immune response of four X-isomers and their effects on plasma cholesterol and triglyceride levels in both C57BL/6J and Apoe−/− mice. The authors showed that the four immunogens produced significant immunogenicity against native PCSK9 to day 120 after immunization of C57BL/6J and Apoe−/− mice. This resulted in significantly decreased plasma cholesterol levels in C57BL/6J mice, and to a lesser degree in Apoe−/− mice. The X-PCSK9-B1 treated mice had increased LDL receptor mRNA and protein levels at day 120 after treatment. Thus, this study provides a new, potentially promising approach that uses long-term immunotherapy for a treatment of hypercholesterolemia.

## 1. Introduction

PCSK9 (Proprotein convertase subtilisin/kexin type 9) is synthesized in the liver and secreted into plasma [1,2]. It acts by binding to the LDL receptor (LDLR) on the cell surface and chaperones the LDLR toward the lysosomal compartment for degradation. Thus, this action of PCSK9 decreases the number of LDLR, which are available to be recycled back to the cell surface to remove LDL from the plasma. Decreased LDLR results in increased plasma LDL levels [2]. A gain-of-function PCSK9 mutation leads to lower amounts of LDLR, hence higher levels of LDL cholesterol [3,4]. To contrast, a loss-of-function mutation of PCSK9 leads to lower levels of LDL cholesterol, resulting in significant reduction in coronary events [5,6].

Significant research effort in the last five years has focused on developing therapeutic agents to decrease PCSK9 levels or to prevent PCSK9 action. Currently, there are several types of PCSK9 inhibitors, including small interfering RNA (siRNA), antisense oligonucleotides (ASO) and monoclonal antibodies. The siRNA is designed to silence PCSK9 mRNA with a single-stranded RNA. This approach shows promise in inhibiting PCSK9 mRNA, decreasing PCSK9 protein and reducing LDL cholesterol levels [7]. ASO are short nucleotides that bind to the mRNA and inhibit mRNA translation to protein. This strategy has shown little progress to date. Conversely, PCSK9 monoclonal antibodies, including Alirocumab (REGN727), Evolocumab (AMG145) and Bococizumb (RN316), are already being used in FDA-approved clinical trials. The Evolocumab FOURIER trial has shown a reduction in LDL cholesterol by 59% after 48 weeks, compared to placebo [8,9]. Similarly, the Bococizumab SPIRE trial has shown a reduction of LDL cholesterol by 56% at 14 weeks, compared to the placebo group [10,11]. The Alirocumab ODYSSEY trial has achieved an approximately 60% reduction of LDL cholesterol after 78 weeks [12,13]. Moreover, the recently published FOURIER trial, testing Evolocumab in 27,564 patients, showed a greater than 15% benefit in outcome [9] and a significant reduction of coronary atherosclerotic plaque volume as determined by ultrasound after 18 months of treatment [14]. The ODYSSEY alirocumab outcomes trial (Sanofi Odyssey Outcomes; https://clinicaltrials.gov/ct2/show/NCT01663402) is a randomized treatment estimated to last five years, and it aims to answer the long-term safety versus benefit of the intervention. Currently, for individual patients, the cost of PCSK9 monoclonal antibody treatment is as high as $14,500 per year. Thus, the high out-of-pocket cost of PCSK9 inhibitors may reduce long-term adherence to the treatment [15]. Due to these findings, searching for a better and more economic therapy is warranted.

To reduce the action of PCSK9 in plasma, the authors propose a novel strategy. Namely, the authors produced a panel of non-native, conformationally-changed isomers of PCSK9 (X-PCSK9) to be used as immunogens, thus developing an active immunotherapy targeting of native PCSK9 and inhibiting/blocking the interaction of PCSK9 with LDLR, which will in turn result in lower plasma cholesterol levels. A disulfide bond-scrambling technique permits reversible conversion of the native and denatured proteins. It can be used to produce stable conformationally changed isomers of unfolded proteins for isolation, characterization and application to clinical usage [16,17]. Accompanying a denaturant and thiol catalyst (0.1 mM β-mercaptoethanol), a disulfide protein will denature and unfold, shuffle its native disulfide bonds, and convert to a mixture of fully oxidized scrambled isomers. These are termed X-isomers. The X-isomers can be captured by HPLC for the characterization of their structures and functions [17]. The laboratory of JY Chang has used this method to show an increased immune response of non-native isomers (X-isomers) to vascular endothelial growth factor (VEGF) in mice [18], demonstrating that X-VEGF isomers are potentially useful compounds for developing active immunotherapy for cancer treatment.

The authors used this scrambled disulfide bond technique to generate conformationally changed isomers of the catalytic domain (residues 152−452) of mouse PCSK9 in this study. The focus was on four X-isomers generated by this technique to study their immune responses and effects on cholesterol and triglyceride levels in both C57BL/6J and Apoe−/− mice. The X-PCSK9 A1, A2, and B1, B2 immunogens produced significant immunogenicity against native PCSK9 until day-120 after immunization of C57BL/6J and Apoe−/− mice. These four immunogens resulted in significantly decreased plasma cholesterol levels in C57BL/6J mice, whereas only X-PCSK9 B1 decreased plasma cholesterol levels in Apoe−/− mice. The X-PCSK9 B1 immunogen also significantly decreased plasma triglyceride levels in both C57BL/6J and Apoe−/− mice. The effect of X-PCSK9 B1 resulted in significantly increased levels of both hepatic LDL receptor mRNA and protein. This study provides a new promise for a long-term, immunotherapy approach to regulate cholesterol levels.

## 2. Results

### 2.1. Expression, Purification and Disulfide Scrambling of Region-1 And -2 of the Catalytic Domain of PCSK9

#### 2.1.1. Expression and Purification

**Figure 1:**
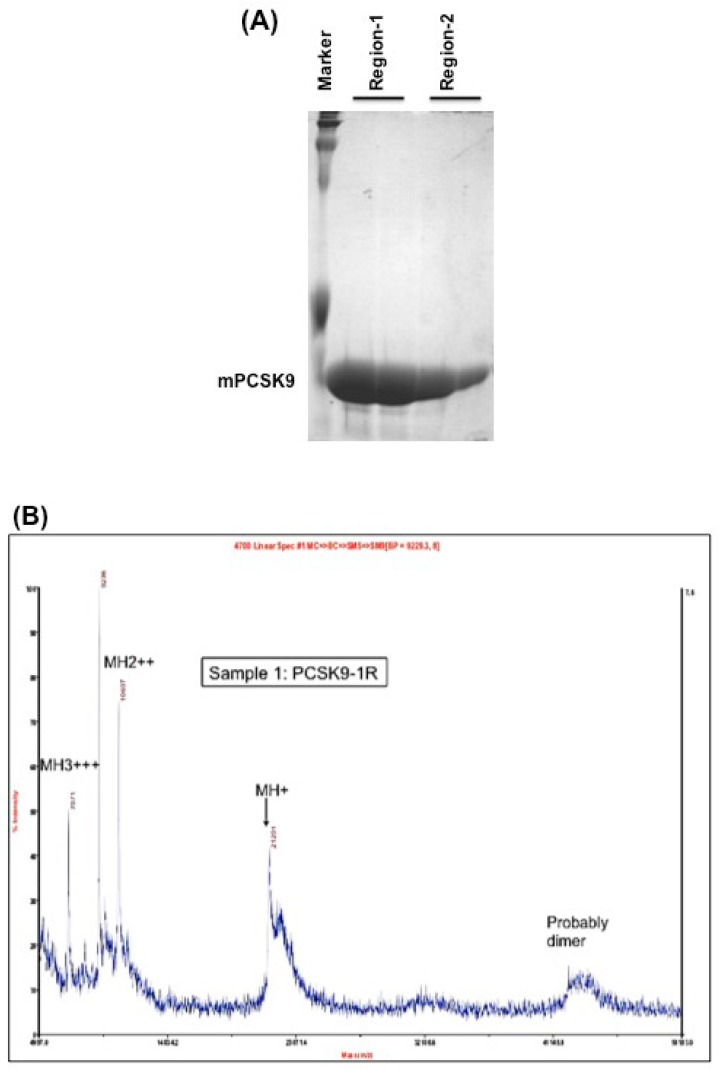

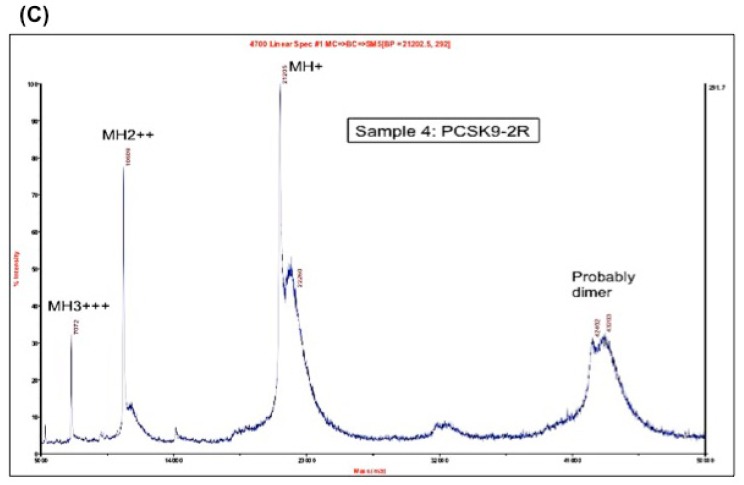
(**A**) Coomassie blue staining of the purified region-1 and region-2 of mPCSK9 proteins. Region-1 (residues 152–351) and region-2 (residues 249–452) of mouse catalytic domain were cloned and expressed by pET-3a plasmids. The proteins were reduced and purified by a reverse-phase high-performance liquid chromatography (HPLC) on an Agilent 1100 HPLC system. The purified proteins were analyzed by SDS/PAGE and stained with Coomassie blue. The positions of expressed proteins are shown; (**B**) MALDI mass spectrometry on Region-1. The molecular mass of purified PCSK9 region-1 in fully reduced form (PCSK9-1R). The molecular mass of PCSK9 region-1 as determined by MALDI was 21,201 Da; (**C**) MALDI mass spectrometry on Region-2. The molecular mass of purified PCSK9 region-2 in fully reduced form (PCSK9-2R). The molecular mass of PCSK9 region-2 as determined by MALDI was 21,355 Da.

Mouse PCSK9 (NP_705793) contains 694 amino acids. The catalytic domain encompasses residues 152–452, which is the main region that interacts with the LDL receptor. The authors chose this region to test the disulfide scrambling technique to generate X-isomers. The authors cloned this region to two overlapping segments: region-1 of mPCSK9 (residues 152–351) contains 4 cysteines and region-2 of mPCSK9 (residues 249–452) contains 6 cysteines to maximize the probability of scrambling the disulfide bonds. These two constructs were cloned into pET-3a expression vector. The proteins were induced by IPTG and purified by HPLC, as described in the Methods section. These two fully reduced and purified protein fragments showed a single band with relatively high purity, as demonstrated by Coomassie blue staining (Figure 1A).

The molecular masses of region-1 and region-2 were determined by MALDI-MS to be 21,201 and 21,355 Da, respectively (Figure 1B,C).

Both proteins showed one peak by MALDI mass spectrometry, which indicates the purity of the proteins. The calculated mass, as determined by MALDI, of both region-1 (21,201 Da) and region-2 (21,355 Da) were close to the theoretical molecular weights (21,201.86 Da and 21,342.56 Da, respectively).

#### 2.1.2. Disulfide Scrambling

**Figure 2:**
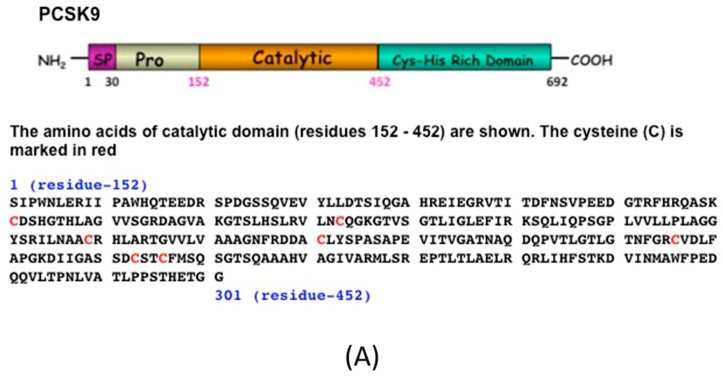

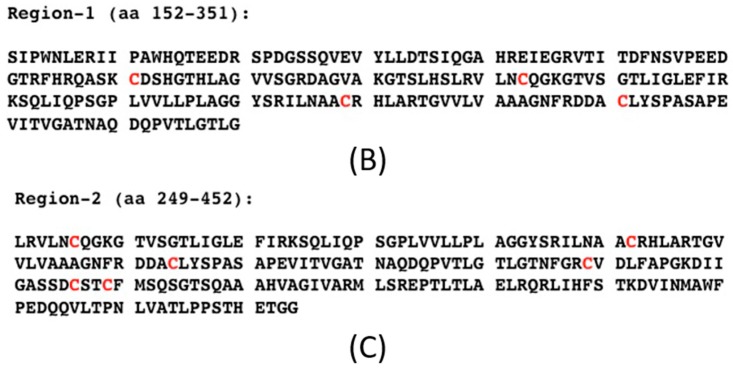
(**A**) Schematic diagram and amino acid sequences of mouse PCSK9. The full-length PCSK9 is shown (amino acids 1–692). The signal peptide (SP) is located at residues 1–30, the prodomain is at residues 31–151, the catalytic domain is at residues 152–452, and the Cys-His rich domain is at residues 453–692. The amino acid sequence of the catalytic domains is shown (aa 152–452); all Cysteines (C) are marked in red. (**B**) The amino acid sequence of region-1 of the mPCSK9 (aa 152–251) contains four Cysteines and should yield three different disulfide isomers as predicted. The theoretical molecular weight is 21,201.86 Da. All Cysteines (C) are marked in red. (**C**) The amino acid sequence of region-2 of the mPCSK9 (aa 249–452) contains six Cysteines and should yield fifteen different disulfide isomers as predicted. The theoretical molecular weight is 21,342.56 Da. All Cysteines (C) are marked in red.

These two reduced proteins, region-1 and region-2, were subjected to disulfide scrambling to generate X-isomers (Figure 2A).

Region-1 contains four Cysteines, which should generate three X-isomers, whereas region-2 contains six Cysteines, which should generate fifteen X-isomers (Figure 2B,C, respectively). These isomers can be separated and collected by HPLC as described by Jui-Yoa Chang [17].

**Figure 3:**
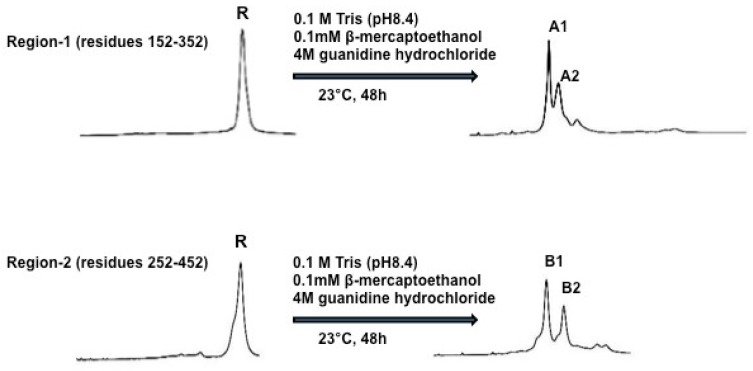
Refolding of Region-1 and Region-2 of PCSK9. Reduced Region-1 and -2 (1 mg/mL) proteins were incubated in 0.1 M Tris-HCl buffer (pH 8.4) containing hydroquinidine chloride (4 M) and β-mercaptoethanol (0.1 mM). The reaction was carried out at 23 °C for 24 h. Samples were analyzed and purified by HPLC using the conditions described in the Methods. The end products were shown to be comprised of two major isomers A1, A2 from Region-1 and B1, B2 from Region-2.

To do oxidative folding and disulfide scrambling of PCSK9, the authors set out to test five different oxidative folding conditions in 0.1 M Tris-HCL pH 8.4. Condition-1 contained 4 M guanidine hydrochloride; condition-2 contained 4 M guanidine hydrochloride plus 0.1 mM β–mercaptoethanol; condition-3 contained 4 M guanidine hydrochloride plus 2 μM CuSO_4_; condition-4 contained 4 M guanidine hydrochloride plus 20 μM Cysteine, and condition-5 contained 4M guanidine hydrochloride plus 20 μM GSSG. Folded samples were acidified with an equal volume of 4% aqueous trifluoroacetic acid and analyzed by HPLC. Following evaluation of the five different conditions, condition-2 (0.1 M Tris-HCl, pH 8.4 plus 4 M guanidine hydrochloride and 0.1 mM β–mercaptoethanol) was chosen to do oxidative folding and disulfide scrambling of PCSK9 for this study, since it provided the best separation of X-isomers. Figure 3 shows the two fully refolded x-isomers of PCSK9 region-1 to A1 and A2 and region-2 to B1 and B2. The molecular masses of folded region-1 and -2 proteins were confirmed by MALDI-MS. The four X-isomers (immunogens), A1, A2, B1 and B2, were used to immunize C57BL/6J and Apoe−/− mice to investigate their effects on plasma cholesterol and triglyceride levels, as well as their effects on hepatic LDL receptor mRNA and protein levels.

### 2.2. X-Pcsk9 Isomers Produced Antibody Against Native Pcsk9 in Mice

**Figure 4:**
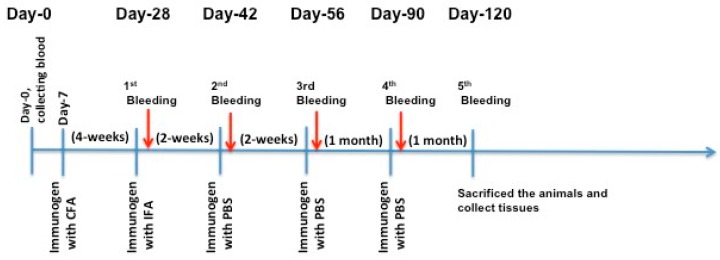
Immunization Strategy. Each mouse (*n* = 5/group) was injected subcutaneously with 100 μL of the immunogen (25 μg/mouse) or PBS in complete Freund’s adjuvant (CFA) as the first injection. Four weeks later, the authors did the first boosting in incomplete Freund’s adjuvant (IFA), followed by injection once a month with X-isomer in PBS for three months. One week after each boost injection, mouse blood was collected for analysis of antibody production titer and cholesterol and triglyceride levels. The authors sacrificed the animals and collected tissues for RNA and protein analyses at the end of the study (day-120).

The authors immunized C57BL/6J and Apoe−/− mice with X-PCSK9 A1, A2, and B1, B2. Control mice were injected with the same volume of PBS. The authors collected blood via retro-orbital bleeding at day 0 before immunization, and days 28, 42, 56, 90 and 120 after immunization (Figure 4). The animals were sacrificed; organs such as liver, spleen, and aorta were collected at the end of the experiment. The collected blood was analyzed for immunogenicity studies, and the measurement of plasma cholesterol and triglyceride levels. Total RNAs in the livers and liver homogenates were used to determine the gene expression and protein levels of LDL receptors, respectively.

**Figure 5:**
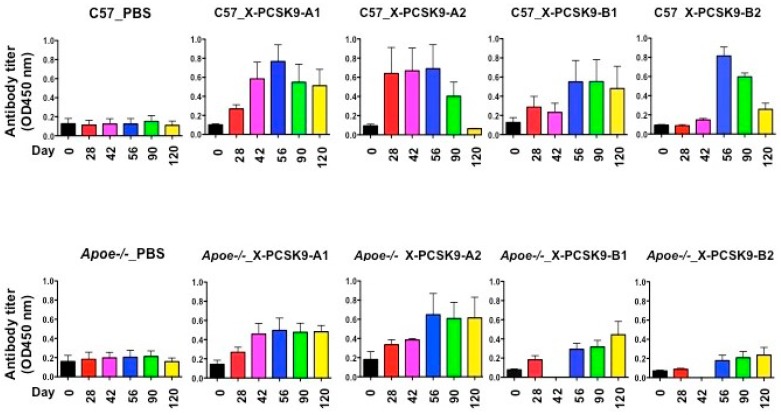
Immunogenicity of X-PCSK9 isomers against native PCSK9 in C57BL/6J and Apoe−/− mice. *C57BL/6J* (*n* = 5/group) and Apoe−/− mice (*n* = 5/group) were immunized with designated X-PCSK9 immunogens A1, A2, and B1, B2 as described in Figure 4. Blood from the animals was collected as described at the indicated time points (black = day-0, red = day-28, pink = day-42, blue = day-56, green = day-90 and yellow = day-120). Plasma was used to determine the antibody titer against native PCSK9 by ELISA. The results are shown as antibody titer (OD450nm) as mean ±SEM. The *p*-value is listed in Table 1.

The immunogenicity of X-PCSK9 A1, A2 and B1, B2 against native PCSK9 increased significantly as a function of time after immunization of *C57BL/6J* and Apoe−/− mice (Figure 5 and Table 1). The immunogenicity of each immunogen increased over 2–8-fold, compared to day-0 before injection. Together, these results show that X-PCSK9 isomers generated antibodies against native PCSK9 in mice.

### 2.3. X-PCSK9 Immunogens Decreased Plasma Cholesterol Levels in C57BL/6J and Apoe−/− Mice

The treatment of immunogens of X-PCSK9-A1, A2 and B1, B2 on plasma cholesterol levels in both C57BL/6J and Apoe−/− mice are presented in Table 2. Regarding C57BL/6J mice, the plasma cholesterol levels are significantly decreased as a function of time from day-0 to day-120 (Table 3). However, only the X-PCSK9-B1 treatment reached a statistically significant level (*p* = 0.0325), compared to PBS treatment. Conversely, in Apoe−/− mice, only X-PCSK9-B1 treatment reached a statistically significant level as a function of time (Table 3, *p* = 0.0069). However, the treatment effect of X-PCSK9 immunogens, A1, A2, and B1, B2, on Apoe−/− mice all produced significantly decreased plasma cholesterol levels (Table 3, treatment effect *p* < 0.0001) compared to the PBS group.

### 2.4. X-PCSK9 Immunogens Decreased Plasma Triglyceride Levels in C57BL/6J and Apoe−/− Mice

The treatment of immunogens of X-PCSK9, A1, A2 and B1, B2, on plasma triglyceride levels in both *C57BL/6J* and *Apoe*−/− mice are presented in Table 4. The treatment of immunogens of X-PCSK9, A1, A2 and B1, B2, in *C57BL/6J* mice showed a significant decrease of plasma triglyceride levels (Table 5, treatment effect *p* < 0.0001), but only X-PCSK9-A1 and -B1 treatment reached a statistically significant level as a function of time (Table 5, *p* = 0.0045, 0.0434, respectively). The treatment effect of immunogens did not reach significant values for plasma triglyceride levels in *Apoe*−/− mice, but the time effects reached statistical significance for all three immunogens, including X-PCSK9-A1, A2 and B1 (Table 5, *p* = 0.0038, 0.0029, 0.0033, respectively). Taken together, the data of the effect of the X-PCSK9 isomers on plasma cholesterol and triglyceride levels demonstrates that X-PCSK9 isomers, specifically X-PCSK9-B1, significantly decrease plasma cholesterol and triglyceride levels.

### 2.5. The Functional Effect of X-PCSK9 in LDL Receptor mRNA: X-PCSK9-B1 Treatment Increased Hepatic LDL Receptor mRNA Level in Both C57BL/6J and Apoe−/− Mice

**Figure 6:**
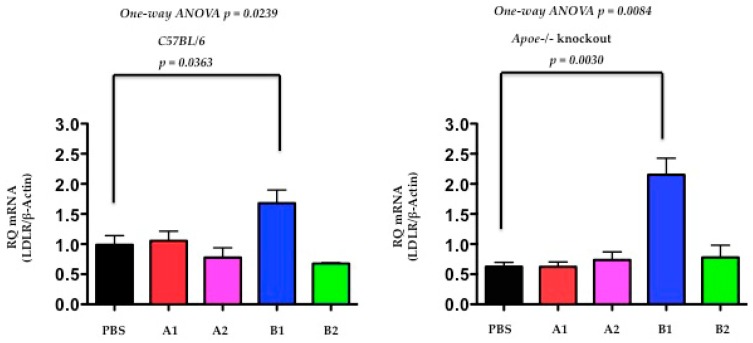
The hepatic mRNA levels of LDL receptor were significantly increased at day 120 after X-PCSK9-B1 treatment in both C57BL/6J and Apoe−/− mice. The authors extracted total RNA from mouse liver at day-120 after X-PCSK9 immunogen treatment. The authors used real-time RT-PCR to quantify the mRNA levels of LDL receptors (LDLR). The results are expressed as RQ (LDLR mRNA normalized with β-Actin; mean ± SEM, black = PBS; red = X-PCSK9-A1; pink = X-PCSK9-A2, blue = X-PCSK9-B1 and green = X-PCSK9-B2). Statistical analyses were performed using two-tailed unpaired *t*-test with Welch’s correction. The differences of X-PCSK9-B1 treated vs. PBS were significantly different in both C57BL/6J and Apoe−/− mice (*p* = 0.0363 and 0.0030, respectively). One-way ANOVA was also performed. The statistical analyses were performed using GraphPad Prism software (version 5). The *p* value < 0.05 is considered significant.

The authors determined the LDL receptor levels by quantitative PCR (qPCR) at day-120 after the treatment of immunogens of X-PCSK9-A1, A2 and B1, B2 in C57BL/6J and Apoe−/− mice. Both C57BL/6J and Apoe−/− mice, had the levels of LDLR mRNA significantly increased after immunogen X-PCSK9-B1 treatment, compared to the PBS-treated group (Figure 6, *p* = 0.0363 and 0.0030, respectively). These results suggest that X-PCSK9-B1 antibody increases LDLR mRNA levels, resulting from the inhibition of the interaction of PCSK9 with LDLR.

### 2.6. The Functional Effect of X-PCSK9 in LDL Receptor Protein: X-PCSK9-B1 Treatment Increased the Levels of Hepatic LDL Receptor Proteins in Both C57BL/6J and Apoe−/− Mice

**Figure 7:**
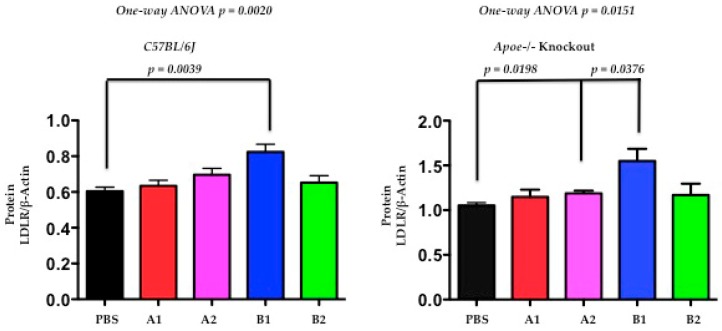
The levels of LDL receptor proteins were significantly increased after X-PCSK9-B1 treatment in both C57BL/6 and Apoe−/− mice and after X-PCSK9-A2 treatment in Apoe−/− mice. We immunized C57BL/6J (males; *n* = 5/group) and Apoe−/− (males; *n* = 5/group) with PBS, X-PCSK9-isomers (A1, A2, B1 and B2) as described in the Methods. At day-120 after treatment, mice were sacrificed, and liver tissues were collected. Liver homogenates (50 μg) were resolved by SDS/10% PAGE, followed by Western blot analysis to determine the levels of LDLR and Actin. The ratios of LDLR/β-Actin of each group are presented as mean ± SEM (black = PBS; red = X-PCSK9-A1; pink = X-PCSK9-A2, blue = X-PCSK9-B1 and green = X-PCSK9-B2). The authors used two-tailed unpaired *t*-test with Welch’s correction to analyze the difference between X-PCSK9 isomers vs. PBS-treated groups. The statistical analyses performed using GraphPad Prism software (version 5). The *p* value < 0.05 is considered significant. One-way ANOVA statistical analysis was also performed using the same software.

Next, the authors used Western blot analysis to determine the levels of hepatic LDLR protein at day-120 after immunogen treatment. The hepatic levels of LDLR protein were significantly increased in both C57BL/6J and Apoe−/− mice (Figure 7, *p* = 0.0039, 0.0376, respectively) after X-PCSK9-B1 immunogen treatment. The X-PCSK9-A2 treatment also significantly increased LDLR protein levels in Apoe−/− mice (*p* = 0.0198). Taken together, our results revealed that X-PCSK9-B1 immunogen treatment increased both LDLR mRNA and LDLR protein levels.

### 2.7. The Functional Effect of X-PCSK9 in Plasma PCSK9 Concentration: X-PCSK9-Isomers Treatment Increased the Levels of Plasma PCSK9 in Both C57BL/6J and Apoe−/− Mice

**Figure 8:**
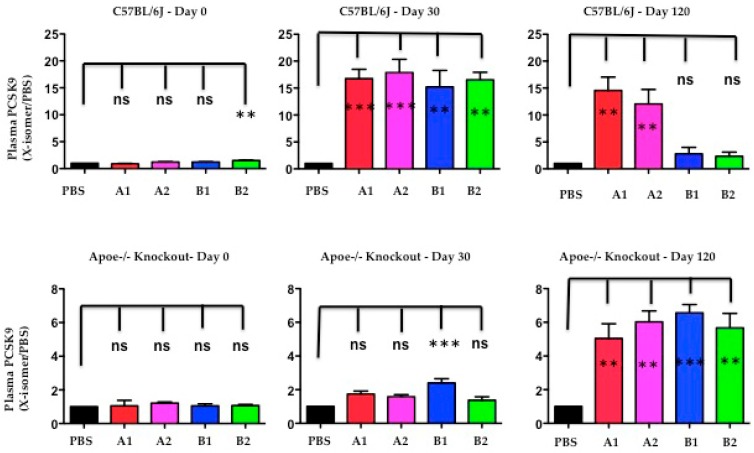
The levels of plasma PCSK9 proteins were significantly induced after immunogen treatment in both C57BL/6 and Apoe−/− knockout mice. Plasma of PBS and isomer treatment group (A1, A2, B1 and B2) at day-0 before treatment, day-30 and day-120 after treatment was subjected to resolve PCSK9 on SDS/10% PAGE, followed by Western blot analysis to determine the levels of PCSK9. The ratios of each treatment group/PBS are presented as mean ± SEM (black = PBS; red = X-PCSK9-A1; pink = X-PCSK9-A2, blue = X-PCSK9-B1 and green = X-PCSK9-B2). The authors used One-way ANOVA statistical analysis with Dunnett’s multiple comparison test to analyze the difference between X-PCSK9 isomers vs. PBS-treated groups. The statistical analyses were performed using GraphPad Prism software (version 5). The *p* value < 0.05 (**, ***) is considered significant.

The authors used Western blot analysis to determine the levels of plasma PCSK9 protein after immunogen treatment at day-30 and day-120. The plasma PCSK9 levels were significantly increased in C57BL/6J mice at day-30 after X-PCSK9-A1, A2, B1 and B2 treatment (Figure 8, *p* < 0.05; increased 17-, 18-, 15- and 17-fold, respectively). The increased levels persisted to day-120 in the groups treated with X-PCSK9-A1 and -A2 (15- and 12-fold, respectively), whereas the PCSK9 levels of X-PCSK9-B1 and -B2 reduced to basal levels (3- and 2-fold, respectively). The immune responses of plasma PCSK9 levels after treatment were less intense in Apoe−/− mice (Figure 8). Only X-PCSK9-B1 treatment group had significantly increased plasma PCSK9 levels to 2-fold at day-30 after. At day-120 after immunogen treatment, the plasma PCSK9 levels of all four groups increased to over 5-fold (Figure 8, *p* < 0.05; 5-, 6-, 7- and 6-fold, respectively).

The induction of increase PCSK9 levels after immunogen treatment was observed in humans treated with monoclonal antibodies (Alirocumab or Evolocumab). The changes in plasma PCSK9 levels varied from no change to a more than 20-fold increase, compared with before treatment [19]. This observation is most likely caused by the delay clearance of the PCSK9-immune complex by immune cells. In this study, by day-120, the plasma PCSK9 levels of B1 and B2 treatment in C57BL/6 mice had decreased to initial levels. Moreover, the slow response of plasma levels in Apoe−/− mice correlated with the less efficient treatment in these mice.

## 3. Discussion

Protein folding from its unfolded state to its fully folded native state is a dynamic process. Many intermediate species are generated along this folding coordinate. The ability to trap and to study the intermediate state of protein folding would provide an understanding of the molecular function of these intermediate structures and the potential nature of misfolding during disease development. The authors used a disulfide scrambling method [17] to produce defined non-native conformational isomers of disulfide proteins (X-isomers), and showed that non-native isomers of vascular endothelial growth factor (VEGF) produced an immune response against native VEGF [18]. These results provide a basis for the development of a vaccine strategy for immunotherapy to treat diseases.

The authors used the strategy of disulfide scrambling of the catalytic domain regions of PCSK9. The authors fractionated four X-PCSK9 isomers (A1, A2 and B1, B2) using HPLC. These four isomers produced significant immune responses toward native PCSK9 for four months in both C57BL/6J and Apoe−/− mice. These results demonstrate that X-PCSK9-A1, A2 and B1, B2 significantly decrease plasma cholesterol levels in C57BL/6J mice by day-120. The X-PCSK9-B1 only decreased plasma cholesterol levels in Apoe−/− mice. These antigens also significantly affected plasma triglyceride levels in both mouse models. Among these four immunogens, X-PCSK9-B1 was most effective in decreasing plasma cholesterol and triglycerides in both C57BL/6J and Apoe−/− mice. Most significantly, X-PCSK9-B1 increased hepatic LDL receptor mRNA and protein levels in bothC57BL/6J and Apoe−/− mice after treatment. Thus, this method provides an alternative way to influence plasma lipid levels for a longer period.

PCSK9 monoclonal antibodies are the most well-developed PCSK9 inhibitors. There are three FDA-approved antibodies: alirocumab (REGN727), Evolocumab (AMG145) and Bococizumab (RN316). Evolocumab, a very effective PCSK9 inhibitor, has been reported to reduce the risk of major cardiovascular events [9]. However, these monoclonal antibodies have to be administered once every two weeks and, thus, represent a very costly and time-consuming regimen [20]. Conversely, a vaccine strategy would be more cost effective for long-term treatment. This study used non-native conformational PCSK9 isomers to generate antibodies against native PCSK9. The authors showed, as proof-of-concept, that immune responses persisted to 120 days. The inhibition of PCSK9 decreased cholesterol and triglyceride levels and increased LDL receptor mRNA and proteins. Unlike the effect of monoclonal antibodies in mice, which showed no effect on the levels of LDL receptor mRNA, but increased LDL receptor protein levels [21], this study noted that mice treated with X-PCSK9-B1 increased both the levels of LDL receptor mRNA and protein. Thus, the increased level of LDL receptor mRNA might result in a long-term effect of decreased lipids levels.

The C57BL/6J wild-type mice have normal lipid levels. Their cholesterols are mostly carried by high-density lipoprotein (HDL) particles. This mouse model has been used to test the initial efficiency of PCSK9 monoclonal antibodies [21]. In contrast, Apoe−/− mice exhibit hyperlipidemia [22], and they have elevated levels of very-low-density lipoproteins (VLDL) and low-density lipoproteins (LDL). Thus, these two models have distinct differences in lipoproteins. The authors thought to use these two different mouse models to investigate the effect of X-PCSK9 isomers on their lipid levels. The effect of PCSK9 in Apoe−/− mice is controversial. Denis M et al. [23] shows that eliminating PCSK9 from Apoe−/− mice decreases plasma cholesterol and VLDL and LDL cholesterol levels. Significantly, these mice accumulated less cholesterol ester in the atherosclerotic plaques, and the plaque sizes were decreased, although this did not reach significant levels. Thus, PCSK9 deficiency protects Apoe−/− mice from developing atherosclerosis. Roche-Molina M et al. [24] used an AAV vector to overexpress PCSK9-D374Y mutants, as a gain-of-function approach in Apoe−/− mice. The results showed synergistic effects on Apoe−/− mice, which developed twice the number of atherosclerotic lesions as non-treated Apoe−/− mice. Thus, PCSK9 exerts a detrimental effect on Apoe−/− mice. In contrast, Tavori H et al. [25] showed that the effects of PCSK9 on lipid and lipoprotein levels were LDLR dependent, but ApoE independent. However, PCSK9 caused Apoe−/− mice to have significantly increased atherosclerotic lesions. Ason B et al. [26] showed that reduction of PCSK9 had no effect on either Ldlr−/− or Apoe−/− mice, thus suggesting that both LDLR and ApoE are required for the function of PCSK9. This study showed that X-PCSK9 treatment had a lesser effect on cholesterol and triglyceride levels in Apoe−/− mice.

Vaccination has been considered to be a safer and more effective long-term treatment for many diseases. Vaccination against the renin-angiotensin system has been developed to lower high blood pressure [27,28,29]. Vaccine against α-synuclein demonstrated a sound rationale for treating Parkinson disease [30,31]. Cancer vaccination has also proven to be a new, effective approach for the treatment of specific cancers [32]. Thus, therapeutic vaccines have the potential to be a good regimen to treat many diseases. Although, in this study, X-PCSK9-B1 isomers significantly decreased cholesterol levels, they did not produce the same marked decrease in cholesterol levels (50% reduced levels) as shown in monoclonal antibody studies against PCSK9. Thus, the authors were able to explore other regions of PCSK9, such as the C-terminal, to compare the stability of X-isomers between the catalytic domain versus the C-terminal of PCSK9. The C-terminal of PCSK9 was found to be essential to induce LDL receptor degradation [33]. An antibody against the C-terminal domain of PCSK9 lowered LDL cholesterol levels in cynomologous monkeys [34].

To summarize, the data presented here provide evidence that an immunogenic vaccine approach using the disulfide scramble method might represent a viable therapeutic approach to produce a long-term treatment for hypercholesterolemia.

## 4. Materials and Methods

### 4.1. Expression and Purification of the PCSK9 Catalytic Domain for Isomer Formation

**Figure 9:**
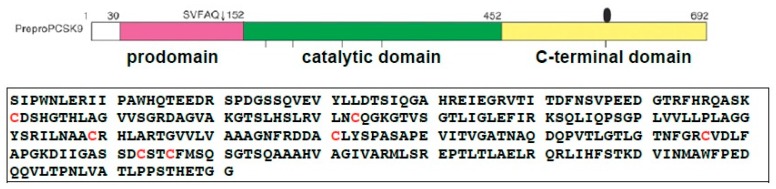
The amino acids sequences of mouse PCSK9 catalytic domain (residues 152-452). All Cysteins (C) are marked in red.

The crystal structure and biochemical studies revealed that catalytic domain (residues 152–452) of PCSK9 binds to the EGF-A domain of the LDL receptor [2]. This region contains 7 Cysteines (in red), which is feasible to scramble the disulfide bonds to generate non-native X-isomers of PCSK9 (Figure 9). 

To scramble the disulfide bonds of this catalytic domain region, the authors chose to express the protein of this region as two overlapping regions, region-1 (residues 152–351, contains 4 Cysteines) and region-2 (residues 249–452, contains 6 Cysteines), to maximize the probability of scrambling the disulfide bonds. The authors cloned and expressed region-1 and region-2 by using pET-3a expression vector (Novagen). The sequences of each construct cloned to the pET-3a plasmid were confirmed by DNA sequencing. Each pET-3a construct was transformed into a Rosetta2 (DE3) *E. coli* strain, which has been shown to enhance protein expression that contains rare codons. The culture was induced using IPTG at 0.75 μM for 6 h at 37 °C with constant shaking. The authors lysed the cell pellets by using 10-mL Sigma cell lytic B cell lysis reagent per 1-gram cell pellet, followed by lysozyme and DNase I incubation. The proteins were solubilized and reduced in 6 M guanidine chloride containing 0.1 M DTT. The authors used a reverse-phase high-performance liquid chromatography (HPLC) on an Agilent 1100 HPLC system (Column ZORBAX 3000 SB-C18, 9.4 mm × 25 cm) to purify the proteins. The buffers used to purify proteins were buffer A containing 0.088% trifluoroacetic acid (TFA) in water and buffer B containing 0.084% TFA in 90% acetonitrile. The peak was collected and subjected to analysis by SDS/12.5 %PAGE using Coomassie blue staining to examine the purity of the proteins. The purified protein was lyophilized and stored at –20 °C until the protein folding step. The molecular mass of the purified PCSK9 was verified by MALDI mass spectrometry. The authors produced 22 mg protein for region-1 and 31 mg protein for region-2.

Full-length mPCSK9 with His tag was cloned into a pcDNA3 expression vector and transfected into 293 F cells (Invitrogen, Carlsbad, CA, USA). The proteins were purified from culture media at 48 h after transfection using His Gravitrap kit (GE health life science, #28401351) according to the instruction from the manufacturer. The purified proteins were dialyzed against water overnight, lyophilized and stored at –20 °C.

### 4.2. Preparation of X-Isomers (X-PCSK9) from the Fully Reduced Proteins Using Oxidative Folding and Disulfide Scrambling

The authors used oxidative folding and disulfide bond scrambling to produce X-isomers. This technique was optimized by Jui-Yoa Chang and his laboratory [16,17,18,35]. The reduced purified region-1 and -2 proteins (1 mg/mL) were incubated in 0.1 M Tris-HCl buffer (pH 8.4) containing hydroguanidine chloride (2 M) and β–mercaptoethanol (0.1 mM) to fold the protein by scrambling their disulfide bonds. The reaction was carried out at 23 °C for 24 h. Folded samples were acidified with an equal volume of 4% aqueous trifluoroacetic acid, and the non-native X-isomers were separated by HPLC using the conditions described above on an Agilent 1100 HPLC system. Each peak was collected, lyophilized and stored at –20 °C. Altogether, the authors generated 2–3 mg of each isomer for immunization from 50 mg of reduced PCSK9 with purity at ~ 90–95 %.

### 4.3. Q-TOF LC/MS

The molecular mass of peptides was determined by Q-TOF mass spectrometer (MS). Briefly, individual PCSK-9 protein conformation (A1, A2, B1, B2) was digested using trypsin or thermolysin as described in the authors’ previous publications [18,36]. The digested samples were analyzed on an Agilent 6538 Ultra High Definition Accurate-Mass Quadrupole Time-of-Flight (Q-TOF) LC/MS system interfaced with an Agilent 1200 Series HPLC-Chip/MS system. The LC-Chip was equipped with a 40 nL enrichment column and an analytical column (75 μm × 43 mm) packed with ZORBAX 300SB-C18 5 μm. The solvents used were 0.1% formic acid in water (A) and 90% acetonitrile in water with 0.1% formic acid (B). The flow rates of sample loading were 3.5 μL/min for the enrichment column and 400 nL/min for the analytical column. Samples were loaded on the enrichment column using 3% solvent B. The gradient for the analytical column was as follows: 3% B at 0 min, 40% B at 9 min, 90% B at 14 min and 3% B at 15 min. The Q-TOF was operated in positive mode with capillary voltage 1800 V and drying gas flow rate of 4 L/min at 340 °C.

### 4.4. Calculations

The authors calculated the molecular weight (MW) of all possible peptides after trypsin (more specific cutting sites) or thermolysin (less specific cutting sites) digestion using methods as outlined in http://web.expasy.org/peptide_cutter/. The molecular masses detected by Q-TOF were compared with those derived via prediction for peptides containing cysteine that could possibly form a disulfide bond. The disulfide bond was identified if molecular masses from experimental and prediction approaches match each other precisely.

### 4.5. Immunization of Male C57BL/6J and Apoe−/− Knockout Mice

Male C57BL/6J and Apoe−/− knockout mice were purchased from Jackson Laboratories and divided randomly into five groups (five mice in each group). The mice were immunized with PBS or four X-PCSK9 isomers (denoted X-PCSK9-A1, X-PCSK9-A2, X-PCSK9-B1, and X-PCSK9-B2). The mice (*n* = 5/group) received vehicle buffer PBS served as a negative control. The immunization protocol consisted first of an injection with proteins (25 μg per 100 μL) with complete Freund’s adjuvant (CFA; Sigma-Aldrich, St. Louis, MO, USA). Two weeks later, mice received another injection of proteins (25 μg per 100 μL) with incomplete Freund’s adjuvant (IFA), followed by boosting once every 2 weeks (for the subsequent one month) with proteins (25 μg per 100 μL) in phosphate-buffered saline (PBS). One week after each boost injection, the authors collected plasma samples for antibody response analysis by ELISA. The authors followed the guidelines of the Animal Protocol Review Committee of the University of Texas Health Science Center at Houston to perform all animal experiments (UTHealth Animal Welfare Committee AWC-15-0182, expiration date 12/31/2018).

### 4.6. Immunogenicity Determination Using ELISA

The authors used ELISA to quantify the antibody levels in plasma. A 96-well MaxiSorp plate (Nunc) was coated overnight at 4 °C with 100 μL/well purified mPCSK9-His (50 ng/mL) expressed in 293 freestyle cells. The authors blocked each well with PBS blocking buffer (Pierce) at 150-μL/well for 1 h. The serially diluted sera samples were then added to each well in duplicate and incubated for 1 h. Each well was the washed with PBS-Tween to remove the unbounded antibody, followed by incubation with goat anti-mouse IgG antibody coupled with horseradish peroxidase (Abcam, Cambridge, MA, USA) (1:5000) for 1 h. Once the reaction ended, TMB peroxidase substrate kit (Pierce) was used to detect the concentration of antibody. Following the final wash, TMB (100 μL/well) was added to incubate for 0.5 h and the reaction was stopped by adding 2 N H_2_SO_4_. The authors used 450 nm absorbance values to detect the reaction which was performed in triplicate. The significance of the results was analyzed using statistical analysis.

### 4.7. Analysis of Plasma Cholesterol and Triglyceride Levels

Plasma total cholesterol (TC) and triglycerides (TG) were determined using the Cholesterol E and the l-type Triglyceride M kits, respectively (Wako Chemicals, Richmond, VA, USA).

### 4.8. Analysis of Hepatic LDL Receptor mRNA and Protein Levels

#### 4.8.1. Real-Time qPCR

Day-120 after immunization saw mouse livers collected, snap-frozen in liquid nitrogen and stored at –80 °C until analysis. Total RNA was extracted from mouse livers using the Qiazol reagent (Qiagen, Valencia, CA, USA), digested with DNasI (Ambion, Austin, TX, USA) and transcribed into cDNA using an Achieve Reverse Transcription kit (Applied Biosystems, Foster City, CA, USA). The authors used an ABI Prism 7900 Sequence Detection System (Applied Biosystems) to quantify RNA. Total RNA was transcribed to cDNA and quantified using SYBR Green PCR Master Mix (Bio-Rad, Hercules, CA, USA). The authors optimized each primer set to eliminate primer-dimer formation. The RNA was normalized with β–Actin mRNA.

The primers for LDLR are mLDLR, forward primer: 5′GTA TGA GGT TCC TGT CCA TC-3′ (1 μM) and reverse primer: 5′-CCT CTG TGG TCT TCT GGT AG-3′ (1 μM).

The primers for β-Actin are forward primer (5′ TGGAATCCTGTGGCATCCATGAAAC (1 μM) and reverse primer (5′ TAAAACGCAGCTCAGTAACAGTCCG (1 μM)).

#### 4.8.2. Western Blot Analysis

Mouse livers were homogenized in 1 ml ice-cold 1X RIPA buffer containing protease inhibitor. Following homogenization, the tissue homogenate was kept on ice for 15 min, followed by centrifugation at 8000× *g* for 10 min at 4 °C. The authors then re-centrifuged the supernatant one more time. This supernatant was kept at –80 °C until analysis. The protein concentration was determined using Bradford assay. The authors applied 50 μg of liver homogenate supernatant to separate LDLR on SDS/10% PAGE. The LDLR was detected using rabbit polyclonal to human LDL receptor antibody crossed reacted to mouse (LS-C146979, LSBio, Seattle, WA, USA, 1:3000 dilution). An Odyssey Infrared Imaging system (Li-COR, Lincoln, NE, USA) was used to quantify the intensity of each protein band.

Mouse plasma (1 μl) at day-0, day-30 and day-120 was applied to SDS/10% PAGE to resolve PCSK9. The PCSK9 was detected using rat polyclonal to mouse PCSK9 (Biolegend, San Diego, CA, USA, 1:1000 dilution). An Odyssey Infrared Imaging system (Li-COR, Lincoln, NE, USA) was used to quantify the intensity of each protein band.

#### 4.8.3. Statistical Analysis

All results are expressed as mean ± SEM. The changes of immunogenicity, plasma cholesterol, or plasma triglyceride levels with time stratified by treatment were analyzed using a linear mixed model including both fixed effects and random effects. Treatment effects of immunogenicity, plasma cholesterol or plasma triglyceride levels were also analyzed using a linear mixed model adjusting for time effects. All the analyses using mixed models were conducted using PROC MIXED procedure of SAS V9.2 (SAS Institute, Inc., Cary, NC, USA). The authors used F-tests to test the significance of time effects or treatment effects. It is statistically significant with a *p*-value smaller than 0.05.

Analysis of variance (ANOVA) was used to compare LDL receptor mRNA and protein levels among different treatment groups on day 120. A *p*-value less than 0.05 was statistically significant. The comparison of LDL receptor mRNA and protein levels between treatment group versus PBS group were performed using GraphPad Prism software (version 5, La Jolla, CA, USA) unpaired *t*-tests with Welch correction with 2-tailed *p*-values. A *p* < 0.05 was statistically significant.

## Figures and Tables

**Table 1:** Statistical analysis of immunogenicity on X-PCSK9 isomers against native PCSK9 in *C57BL/6J* and Apoe−/− knockout mice. Statistical analyses of all samples were performed using a linear mixed model including both fixed effects and random effects. The *p*-values of time effect on each treatment group are listed here. The *p* < 0.05 are considered significant.

**C57BL/6J Mice**					
Treatment	PBS	X-PCSK9-A1	X-PCSK9-A2	X-PCSK9-B1	X-PCSK9-B2
Time effect (*p*-value)	0.9025	0.0002	<0.0001	0.0061	0.0006
**Apoe−/− Mice**					
Treatment	PBS	X-PCSK9-A1	X-PCSK9-A2	X-PCSK9-B1	X-PCSK9-B2
Time effect (*p*-value)	0.0007	0.0002	0.001	<0.0001	0.0007

**Table 2:** Plasma cholesterol levels in *C57BL/6J* and *Apoe−/−* mice after X-PCSK9 isomers A1, A2, B1 and B2 immunogen treatment. Each value represents the average fasting plasma concentration of 5 mice at the indicated time. The results are expressed as mean ± S.D. ND = not determined.

**C57BL/6J Cholesterol (mg/dL)**	**Day-0**	**Day-28**	**Day-42**	**Day-56**	**Day-90**	**Day-120**
PBS	58 ± 13	63 ± 17	66 ± 12	56 ± 5.1	60 ± 6.1	63 ± 9.3
X-PCSK9-A1	60 ± 9.4	56 ± 7.6	50 ± 7.2	46 ± 4.5	50 ± 9.1	56 ± 12
X-PCSK9-A2	68 ± 5.1	59 ± 5.0	62 ± 6.0	58 ± 7.2	59 ± 7.6	61 ± 4.2
X-PCSK9-B1	66 ± 9.6	64 ± 6.3	71 ± 6.9	61 ± 3.8	59 ± 14	57 ± 12
X-PCSK9-B2	66 ± 3.8	61 ± 4.5	61 ± 1.8	62 ± 2.1	62 ± 3.4	65 ± 2.6
**Apoe−/− Cholesterol (mg/dL)**	**Day-0**	**Day-28**	**Day-42**	**Day-56**	**Day-90**	**Day-120**
PBS	270 ± 39	391 ± 46	403 ± 48	410 ± 98	467 ± 128	471 ± 128
X-PCSK9-A1	342 ± 50	384 ± 36	365 ± 63	348 ± 61	361 ± 69	342 ± 66
X-PCSK9-A2	358 ± 60	393 ± 58	362 ± 79	312 ± 50	304 ± 55	303 ± 62
X-PCSK9-B1	406 ± 64	357 ± 36	ND	335 ± 42	350 ± 58	340 ± 70
X-PCSK9-B2	357 ± 80	358 ± 43	ND	412 ± 38	333 ± 53	321 ± 57

**Table 3:** Statistical analyses of the time and treatment effects of X-PCSK9 isomers, A1, A2, B1 and B2, on plasma cholesterol in *C57BL/6J* and Apoe−/− mice.

**C57BL/6J Mice** **(Cholesterol)**	**PBS**	**X-PCSK9-A1**	**X-PCSK9-A2**	**X-PCSK9-B1**	**X-PCSK9-B2**
Time effect (*p*-value)	0.6978	0.0003	0.0044	0.0014	0.0114
		A1 vs. PBS	A2 vs. PBS	B1 vs. PBS	B2 vs. PBS
Treatment effect (*p*-value)		0.0612	0.9665	0.0325	0.7035
**Apoe−/− Mice** **(Cholesterol)**	**PBS**	**X-PCSK9-A1**	**X-PCSK9-A2**	**X-PCSK9-B1**	**X-PCSK9-B2**
Time effect (*p*-value)	0.0006	0.5566	0.1701	0.0069	0.7087
		A1 vs. PBS	A2 vs. PBS	B1 vs. PBS	B2 vs. PBS
Treatment effect (*p*-value)		<0.0001	<0.0001	<0.0001	<0.0001

The statistical analyses of all samples were performed using a linear mixed model including both fixed effects and random effects. The *p*-values of time effect on each treatment group are listed here. The *p* < 0.05 is considered significant.

**Table 4:** Plasma triglyceride levels in C57BL/6J and Apoe−/− mice after X-PCSK9 isomers A1, A2, B1 and B2 immunogen treatment. Each value represents the average fasting plasma concentration of 5 mice at the indicated time. The results are expressed as mean ± S.D. ND = not determined.

**C57BL/6J Triglyceride (mg/dL)**	**Day-0**	**Day-28**	**Day-42**	**Day-56**	**Day-90**	**Day-120**
PBS	12 ± 5.2	14 ± 5.5	13 ± 2.4	16 ± 2.7	13 ± 10	9.5 ± 3.2
X-PCSK9-A1	51 ± 33	25 ± 4.9	24 ± 9.5	28 ± 7.1	25 ± 11	19 ± 4.7
X-PCSK9-A2	30 ± 8.6	26 ± 2.5	53 ± 15	24 ± 5.6	24 ± 6.5	17 ± 2.0
X-PCSK9-B1	28 ± 11	43 ± 13	22 ± 7.0	39 ± 14	30 ± 10	24 ± 8.0
X-PCSK9-B2	26 ± 10	39 ± 11	17 ± 3.4	26 ± 2.3	29 ± 6.5	22 ± 5.9
**Apoe−/− Triglyceride (mg/dL)**	**Day-0**	**Day-28**	**Day-42**	**Day-56**	**Day-90**	**Day-120**
PBS	55 ± 13	59 ± 18	54 ± 19	40 ± 9.4	49 ± 3.4	37 ± 5.5
X-PCSK9-A1	64 ± 19	90 ± 33	75 ± 30	45 ± 17	50 ± 18	45 ± 19
X-PCSK9-A2	85 ± 30	68 ± 18	69 ± 28	40 ± 19	41 ± 27	43 ± 19
X-PCSK9-B1	82 ± 13	73 ± 21	ND	41 ± 5.5	48 ± 8.1	54 ± 9.9
X-PCSK9-B2	71 ± 25	55 ± 17	ND	67 ± 13	61 ± 9.2	40 ± 13

**Table 5:** Statistical analyses of the time and treatment effects of X-PCSK9 isomers, A1, A2, B1 and B2, on plasma triglyceride levels in *C57BL/6J* and Apoe−/− mice. The statistical analyses of all samples were performed using a linear mixed model including both fixed effects and random effects. The *p*-values of time effects on each treatment group are listed here. The *p* < 0.05 is considered significant.

**C57BL/6J Mice (Triglyceride)**	**PBS**	**X-PCSK9-A1**	**X-PCSK9-A2**	**X-PCSK9-B1**	**X-PCSK9-B2**
Time effect (*p*-value)	0.3284	0.0045	0.1516	0.0434	0.4195
		A1 vs. PBS	A2 vs. PBS	B1 vs. PBS	B2 vs. PBS
Treatment effect (*p*-value)		<0.0001	<0.0001	<0.0001	<0.0001
**Apoe−/− Mice (Triglyceride)**	**PBS**	**X-PCSK9-A1**	**X-PCSK9-A2**	**X-PCSK9-B1**	**X-PCSK9-B2**
Time effect (*p*-value)	0.0756	0.0038	0.0029	0.0033	0.1784
		A1 vs. PBS	A2 vs. PBS	B1 vs. PBS	B2 vs. PBS
Treatment effect (*p*-value)		0.5777	0.7993	0.5533	0.5051
